# 851. Metabolic Changes Contributing to Age-Associated Antifungal Tolerance in *Cryptococcus neoformans*

**DOI:** 10.1093/ofid/ofad500.896

**Published:** 2023-11-27

**Authors:** Kyungyoon Yoo, Natalia Kronbauer de Oliveira, Somanon Bhattacharya, Bettina F Fries

**Affiliations:** Renaissance School of Medicine at Stony Brook University, Stony Brook, New York; Stony Brook University, Stony Brook, New York; WuXi Advanced Therapies, Philadelphia, Pennsylvania; Renaissance School of Medicine at Stony Brook University, Stony Brook, New York

## Abstract

**Background:**

*Cryptococcus neoformans* (CN) is a facultative intracellular pathogen that divides asymmetrically, resulting in different phenotypes of the mother and daughter cells. After 8-10 divisions, mother cells become more resistant to phagocytic killing by macrophages and resistant to antifungals. These resilient traits allow older CN cells to accumulate in the host during chronic infection. Across different pathogenic fungal species, age-associated mitochondrial changes help the cell adapt to various physiological stresses by altering metabolic regulation and ultimately promoting antifungal resistance.

Cryptococcus neoformans is the leading cause of fungal meningitis among immunocompromised populations

**Figure:**
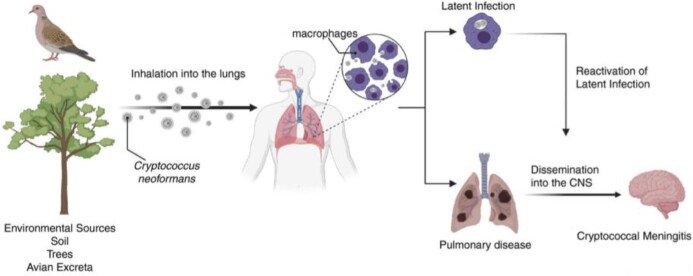

**Methods:**

Ten GEN “old” CN cells were obtained by magnetic bead-based isolation and compared to 1-3 GEN “young” cells. Mitochondrial membrane potential, mass, reactive oxygen species (ROS), calcium, and morphology were evaluated by measuring the fluorescence of appropriate cellular dyes and fluorescent microscopy. ATP level was measured by using a kit measuring fluorescence. Lipid droplets and membrane sterols were quantified and visualized by using appropriate cellular dyes. Ergosterol and sphingolipid levels were quantified by a targeted lipidomic approach using LC-MS-MS.

Old mother cells can be separated and isolated by biotinylation and magnetic sorting

**Figure:**
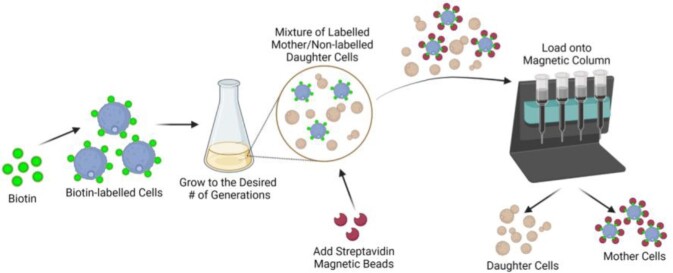

**Results:**

Our data demonstrate old CN cells exhibit increased mitochondrial mass, calcium and ROS levels and decreased mitochondrial membrane potential. Additionally, while the mitochondria from young cells show the formation of tubular networks, the mitochondria of old cells exhibit fragmentation. Furthermore, old CN cells have >5-fold higher ATP levels, which can fuel drug efflux pumps. Old cells show more lipid droplets that localize to the vacuole compared to young cells, which can serve as a cellular source of ATP. RT-qPCR analysis and lipidomic studies indicated changes in the synthesis of various ergosterols and sphingolipids, key lipids that are directly linked to pathogenesis and virulence mechanisms in CN.

Metabolic changes in old Cn cells contribute to antifungal tolerance

**Figure:**
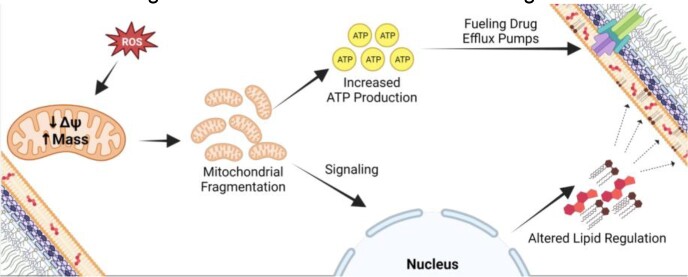

**Conclusion:**

Our findings suggest that mitochondrial dysfunction of older cells is initially beneficial for CN as it promotes a metabolic switch to fatty acid oxidation and altered regulation of various classes of lipids. Increases in ATP production fuel efflux pumps and the export of azoles. This work furthers our understanding of the “old” CN cell phenotype that emerges during aging.

**Disclosures:**

**All Authors**: No reported disclosures

